# Effects of aquaculture effluents on the slender sea pen *Virgularia mirabilis*

**DOI:** 10.1038/s41598-024-59613-3

**Published:** 2024-04-24

**Authors:** Bastien Taormina, Tina Kutti, Siri Aaserud Olsen, Pål Næverlid Sævik, Rita Hannisdal, Vivian Husa, Erwann Legrand

**Affiliations:** Institute of Marine Research, Nordnesgaten 50, 5005 Bergen, Norway

**Keywords:** Environmental impact, Marine biology

## Abstract

This study aims to assess in situ the impact of effluents originating from an Atlantic salmon (*Salmo salar*) farm on a nearby slender sea pen (*Virgularia mirabilis*) field. We evidenced (1) the presence and persistence of emamectin residues (*i.e*. a common chemotherapeutants used for treating ectoparasites in salmons) in *V. mirabilis* tissue 56 days after treatment and (2) lethal and sublethal responses of *V. mirabilis* to effluents discharged by the salmon farm. Particularly, sea pens near the fish farm exhibited significant overproduction of mucus, contraction of polyps’ tentacles, and disappearance of associated fauna. Furthermore, sea pens located directly underneath the farm showed substantial tissue necrosis and, in the most severe case, complete tissue loss and mortality. Our results suggest that lethal damages on sea pens occur directly below the farm, and that sublethal effects are visible up to 500 m from the farm. However, the presence of *V. mirabilis* below the studied farm, which has been active for more than twenty years, suggests that *V. mirabilis* population possesses the capacity to recover from the impacts of the farm, thereby preventing the complete disappearance from the area. In this context, it would be particularly interesting to run a temporal survey following the health state of *V. mirabilis* during an entire production cycle to have a more precise overview of fish farm impacts on this species, including during and after the post-production fallowing period.

## Introduction

Over the last few decades, Atlantic salmon (*Salmo salar*) aquaculture industry has undergone substantial worldwide expansion, with global annual production increasing from 12,000 tonnes in 1980 to 2.7 million tonnes in 2021^1^. Norway is the world’s largest producer of Atlantic salmon with more than half of the world’s production (*i.e.* approximately 1.5 million tonnes in 2021) produced at more than 900 fish farms along the Norwegian coastline^2^.

Atlantic salmon aquaculture constitutes an important source of environmental pressure for surrounding marine ecosystems^3–6^. Particularly, fish production in open sea cages results in the release of organic matter (*i.e.* uneaten food and fish faeces) into the surrounding environment^6–9^. It has been estimated that for every 100 kg of fish feed administered, 10 kg are uneaten while 22.5 kg of fish faeces are produced^10^. Thus, a large-capacity farm (*e.g.* approximately 9000 tonnes of salmon produced per year) can discharge up to 10 tonnes of fish faeces per day during peak production^9,11^. These organic wastes are sinking to the seafloor and disperse over an area, typically from several hundred up to 1000 m away from the farms, dependent on hydrodynamic properties of the site^12^. Particulate organic matter (POM) sedimentation near aquaculture farms can thus reach 8–20 times the natural sedimentation rate^11,13^. Several studies have shown that organic enrichment has adverse impacts on benthic communities in close proximity to the farms^11,14–18^. At the individual scale, high sedimentation rates may cause smothering and burial^19^, metabolic depression^16,18^ and changes in behaviour^20–22^ eventually leading to impacts on taxa survivability^17^. Nonetheless, major knowledge gaps persist on the response of benthic species and their sensitivity threshold to POM effluents coming from fish farms^23^. In addition to organic matter effluents, the release of chemotherapeutants used for the treatment of salmons ectoparasites (*i.e.* sea lice from genus *Lepeoptheirus* and *Caligus*) is also of particular concern. Among these chemotherapeutants, emamectin benzoate (EMB) is a semi-synthetic drug belonging to the avermectin group and is widely used worldwide. EMB is administrated as in-feed additive to salmons and can thus be released into the marine environment via uneaten food and faeces^24^. Although the toxicity of EMB has been proven in various non-target crustacean species (*e.g.* alteration of mortality, moulting behaviour, gene expression, reproduction^25–29^), an important knowledge gap exists concerning its impact on other taxonomic groups, as well as its potential persistence in tissues^30–33^.

Sea pens are colonial octocorals belonging to the superfamily Pennatuloidea. In Norway, five main species of sea pens are present: (1) *Halipteris finmarchina* (Sars, 1851), (2) *Kophobelemnon stelliferum* (Müller, 1776), (3) *Funiculina quadrangularis* (Pallas, 1766), (4) *Pennatula phosphorea* (Linnaeus, 1758) and (5) *Virgularia mirabilis* (Müller, 1776). *V. mirabilis* can form dense fields in muddy and sandy bottoms (*ca* 5 colonies m^–2^) at depth of 10–400 m and is widespread along most of the Norwegian coast^34^. Their morphology introduces structural complexity to otherwise flat and featureless areas, facilitating the creation of new habitats. Thus, these sea pen fields serve as shelter, feeding grounds, and nursery areas for a diverse range of organisms^35–37^. However, sea pen habitats face various threats caused by human activities, such as intense demersal fisheries (*i.e.* trawling) and marine pollution^38^. Thus, OSPAR Commission has recognized sea pen field habitats as threatened and declining, further highlighting their ecological significance^38^. However, sea pen fields are presently not listed as threatened or declining habitat in Norway (Norsk rødliste for naturtyper, artsdatabanken.no), and no protective management measures are applied when spatial overlaps between sea pen fields and Atlantic salmon farms are found. There is a notable knowledge gap regarding the effects of aquaculture on various species of sea pens, which partly explains the lack of management measures in place. Thus, for a better management of this ecologically important habitat, better information on the potential impacts of aquaculture effluents on sea pens is needed.

In this context, the objective of this study is to evaluate, in situ, the impact of effluents from an Atlantic salmon farm on a nearby *V. mirabilis* field. Using video recordings taken along a gradient from one fish farm, we assessed visually the health state of the sea pen colonies. In addition, the concentration of EMB residue, measured as emamectin B1a (*i.e*. the main component of emamectin ≥ 90%), was measured in *V. mirabilis* colonies sampled at different distances from the farm. Finally, these observations were put in relation with environmental variables and POM sedimentation models based on the farm’s production data, to define potential correlations and threshold values.

## Results

### Site characterisation

**Figure 1:**
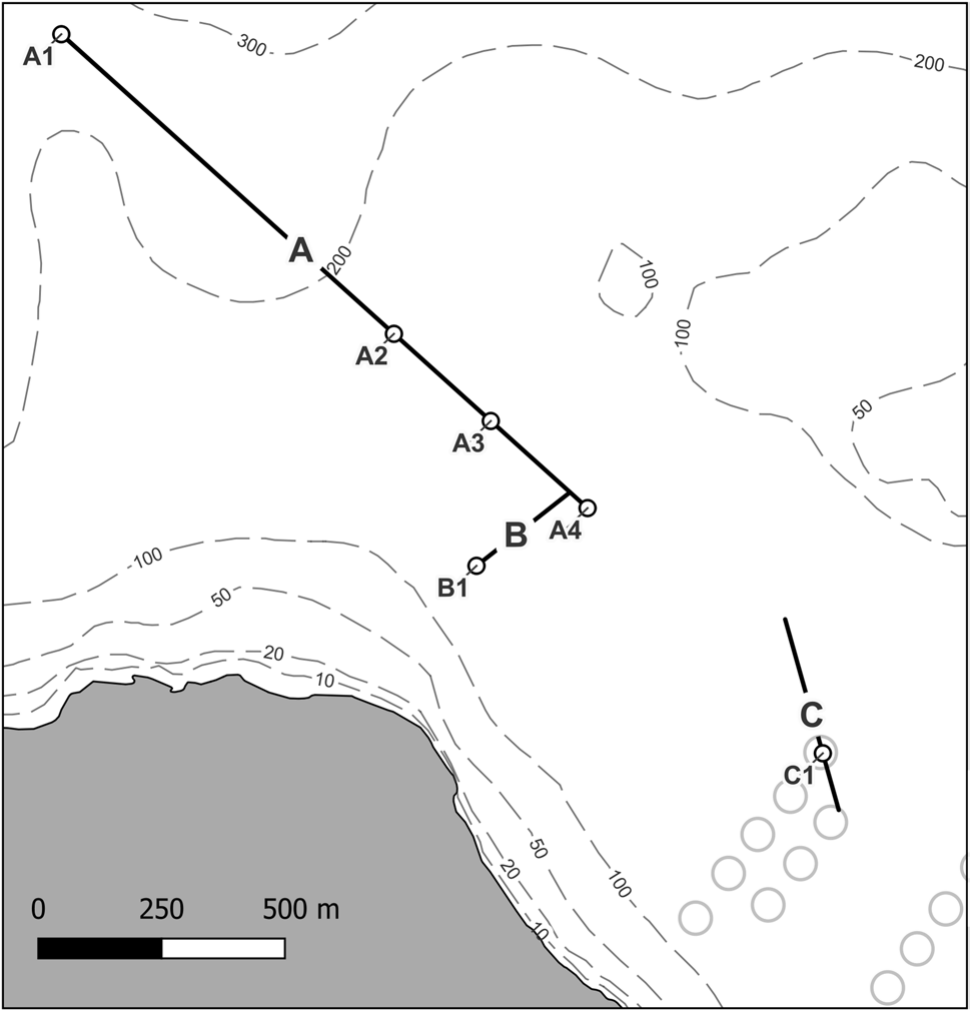
Location of the study site. The three transects surveyed (A, B and C) as well as the different samples locations (A1, A2, A3, A4, B1 and C1) are showed. The location of net cages of the Atlantic salmon farm is presented as grey circles in the bottom-right of the map based on satellite images. (Map created by author using QGIS 3.22.7 software: https://qgis.org/).

**Figure 2:**
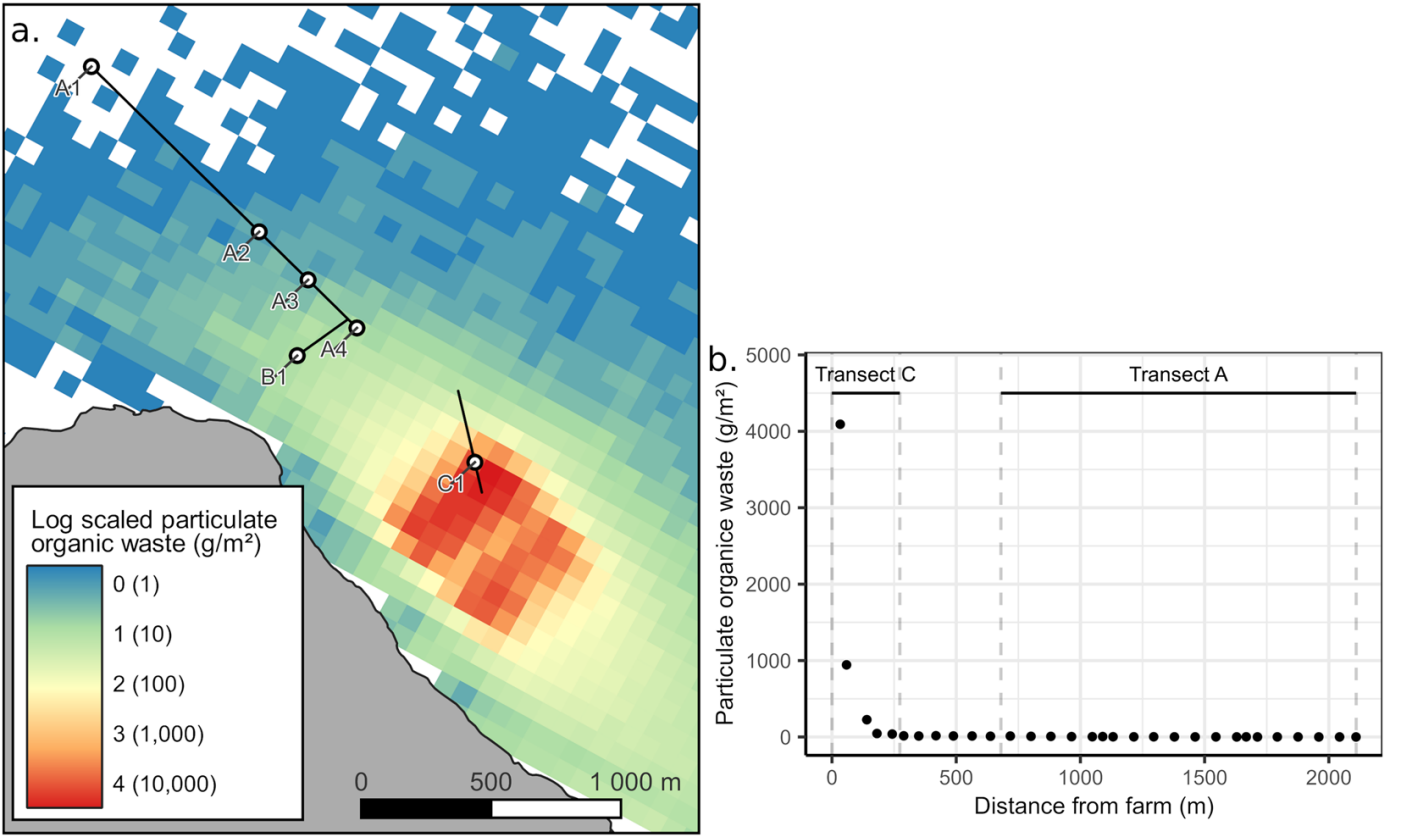
Dispersion model of total particulate organic waste coming from the fish farm between 01/08/2022 and 07/01/2023. (**a**) Total particulate organic waste sedimentation over the study site. (Map created by author using QGIS 3.22.7 software: https://qgis.org/). (**b**) Details of the particulate organic waste sedimentation over transects A and C.

No differences in POM and NO_2_^−^ were observed in the bottom water at sites A1, A2, A3, A4 and B1 (680–2100 m away from the farm; Fig. 1), while NH_4_^+^, NO_3_^−^, PO_4_^3−^ and SiO_4_^4−^ presented minor variations (Table 1). Higher NH_4_^+^, NO_3_^−^, PO_4_^3−^ and SiO_4_^4−^ concentrations were measured at site A1 despite being the furthest from the farm, suggesting that these differences might be due to the deeper location of this site and are thus natural. Organic content in sediment was slightly elevated at sites A2 and A3. Nevertheless, it was below 5% at all sites, which represents background values^11^. Residues of EMB (measured as emamectin B1a, the marker residue of EMB^39^) was below quantifiable levels for all sites. According to the dispersion model, organic waste from the fish farm (*i.e.* total waste sedimentation over the 5 months period preceding our survey) is high straight underneath the production rings (with a maximum value of 9951 g m^−2^) and decrease quickly with increasing distance to the farm (Fig. 2). The modeled sedimentation rates of particulate organice matter at site C1 (below the farm) is estimated to be of 4093 g m^−2^, while at 100 m from the farm this value dropped to 226–38 g m^−2^ at 200 m and to 12 g m^−2^ at 680 m which corresponds to the site A4 (Fig. 2). At a distance greater than 750 m, the total waste deposal is below 10 g m^−2^, and below 1 g m^−2^ 1400 m from the farm.

**Table 1:** Mean ± standard deviation of the different chemical variables measured in bottom seawater (*i.e.* Particulate Organic Matter (POM), NH_4_^+^, NO_2_^−^, NO_3_^−^, PO_4_^3−^, SiO_4_^4−^) and sediment (*i.e.* emamectin B1a (EmaB1a) *i.e.* the main component of emamectin ≥ 90%, and organic content); n = 3.

Site	Distance from farm (m)	Depth	POM (g L^−1^)	NH_4_^+^ (µmol L^−1^)	NO_2_^−^ (µmol L^−1^)	NO_3_^−^ (µmol L^−1^)	PO_4_^3−^ (µmol L^−1^)	SiO_4_^4−^ (µmol L^−1^)	EmaB1a (µg kg^−1^)	OM (g kg^−1^)
A1	2100	230	0.8 ± 0.04	0.06 ± 0.09	0.05 ± 0	10.83 ± 0.06	0.89 ± 0.01	7.47 ± 0.02	< LOQ	2.8 ± 0.27
A2	1200	180	0.72 ± 0.03	0.01 ± 0.01	0.07 ± 0.01	9.42 ± 0.01	0.78 ± 0	6.38 ± 0.04	< LOQ	4.4 ± 0.26
A3	940	180	0.79 ± 0.1	0.03 ± 0.04	0.06 ± 0	8.61 ± 0.04	0.72 ± 0	5.71 ± 0.02	< LOQ	3.8 ± 0.18
A4	680	170	0.74 ± 0.1	0.04 ± 0.03	0.06 ± 0	8,41 ± 0.08	0.69 ± 0	5.62 ± 0.09	< LOQ	2.7 ± 0.35
B1	790	170	0.74 ± 0.07	0.02 ± 0.01	0.06 ± 0	9.23 ± 0.03	0.76 ± 0	6.11 ± 0.05	< LOQ	NA
C1	0	180	NA	NA	NA	NA	NA	NA	NA	NA

Limit of quantification (LOQ) for EmaB1a was 1.0 µg kg^−1^.

### Presence of emamectin in *V. mirabilis* tissue

Residues of EMB were measured as emamectin B1a, the marker residue of EMB^39^. Concentrations measured in each sea pen tissue are presented in Table 2. Emamectin B1a wasn’t detected in any colony coming from site A1, A2, A3 and A4 (*i.e.* sites more than 680 m away from the farm; Fig. 1). Due to the different size and weight of the sea pens, limit of quantification (LOQ) was colony-dependant and varied between 2 and 6 μg kgDW^−1^ (except one sea pen from site A4 with a limit of quantitification of 10 μg kgDW^−1^). Nevertheless, quantifiable levels of emamectin B1a were detected for three sea pens from site C1 (*i.e.* below the farm), with a mean concentration of 8.7 ± 0.5 μg kgDW^−1^, n = 3 (Table 2). The two remaining colonies from this site didn’t show any detectable levels of emamectin B1a, but LOQ was high (LOQ of 15 and 20 μg kgDW^−1^ respectively).

**Table 2:** Concentration of emamectin B1a (EmaB1a, *i.e.* the main component of emamectin ≥ 90%) measured in *Virgularia mirabilis* tissue sampled at different sites (sites A1, A2, A3, A4 and C1).

Site	A1	A2	A3	A4	**C1**
Distance from farm (m)	2110	1200	940	680	0
EmaB1a µg kg^−1^					
Colony 1	< LOQ (3 µg kg^−1^)	< LOQ (6 µg kg^−1^)	< LOQ (4 µg kg^−1^)	< LOQ (2 µg kg^−1^)	8.96
Colony 2	< LOQ (2 µg kg^−1^)	< LOQ (3 µg kg^−1^)	< LOQ (4 µg kg^−1^)	< LOQ (2 µg kg^−1^)	9.11
Colony 3	< LOQ (4 µg kg^−1^)	< LOQ (4 µg kg^−1^)	< LOQ (1 µg kg^−1^)	** < LOQ (10 µg kg**^**−1**^**)**	8.01
Colony 4					** < LOQ (15 µg kg**^**−1**^**)**
Colony 5					** < LOQ (20 µg kg**^**−1**^**)**

Concentrations are expressed in µg kg^−1^ of dry weight (DW). Bold indicated a limit of quantification (LOQ) higher or equal to 10 µg kg^−1^.

### *V. mirabilis* answer to aquaculture

A total of 385 V*. mirabilis* colonies (hereafter simplified to “sea pen”) were observed, evenly distributed along the three video transects (see Supplementary Fig. S1). Of these 385 sea pens, 265 (69%) did not show any signs of reduced health (*i.e.* tissue necrosis or mucus production), 71 (18%) presented elevated mucus production, 72 (19%) presented necrosis, and 16 (4%) presented both mucus production and necrosis. Also, 125 (32%) seapens had contracted their polyps and 132 (34%) had an associated fauna.

**Figure 3:**
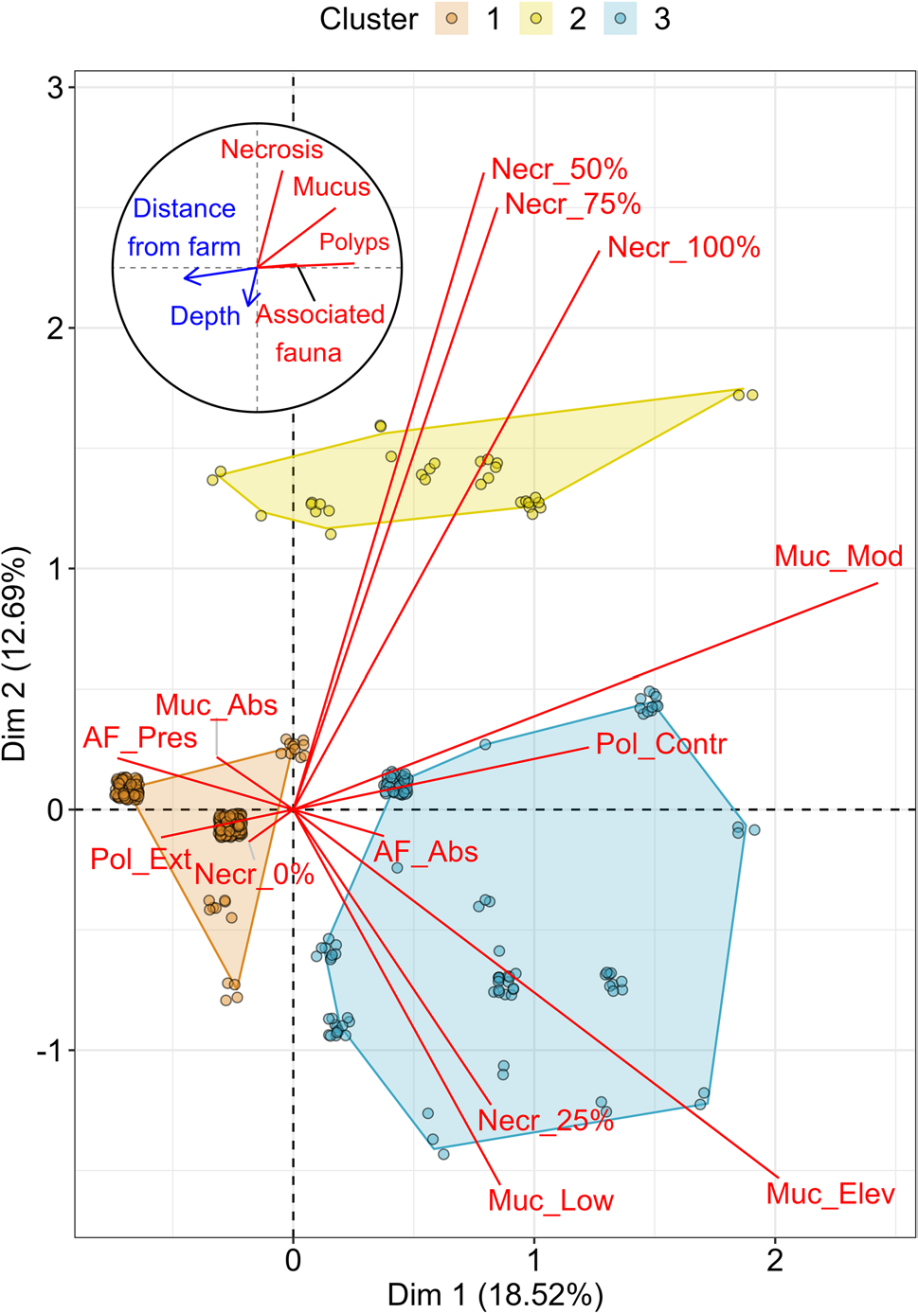
Multiple correspondence analysis ordination of *Virgularia mirabilis* visual health and morphology. Dimensions 1 and 2 represents 31.2% of the total inertia. Vector overlays (red lines) show how modalities of each visual category correlates with the two first axis described (Necr = Necrosis, Muc = Mucus, Pol = Polyps, AF = Associated Fauna; see Table 4 for complete abbreviations used). Correlation circle overlay in the top left shows how visual categories (red lines) and supplementary quantitative variables (blue arrows) correlates with the two first dimensions. Colours represents results of clustering using Ward linkage. Each point represents a *V. mirabilis* colony. In order to have a better view of overlapping points, jittering has been used.

**Figure 4:**
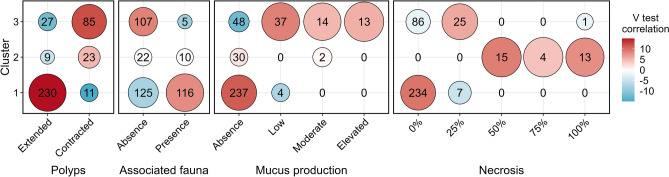
Bubble plot representing contribution of each visual health and morphology modalities for the three different clusters of *Virgularia mirabilis* (see Fig. 3 for clusters definition). Number over each bubble represent the number of colony belonging to this cluster/modality combination. Bubble size represents the ratio of *V. mirabilis* colonies from a cluster belonging to a particular a modality and the number of *V. mirabilis* colonies all clusters combined belonging to the same modality. Colour of each bubble represents the v-test correlation value: it can be seen as a “normalized” difference between the number of colonies from a clusters belonging to a modality and the mean number of colonies per cluster for the same modality. Positive value signifies an over-representation of a modality in a cluster while a negative value signifies an under-representation of this modality in the cluster (see Husson et al.^40^).

Based on the visual description of sea pens, a Multiple correspondence analysis (MCA) ordination has been computed (Fig. 3). The first two axes of this ordination captured 31.2% of the total variation. Overall, “Polyp” and “Associated fauna” categories were correlated with first axis, “Necrosis” category was correlated with second axis while “Mucus production” category seemed to be equally correlated with both axes. Distance from farm of each colony had a strong negative correlation with the first axis while depth had a weak, but existing, correlation with the second axis (Fig. 3). Hierarchical clustering allowed the creation of three different clusters of sea pens based on their visual description, hereafter called cluster 1, 2 and 3 (Fig. 3). The characteristics of each cluster are presented in Fig. 4. Cluster 1 was the most represented, with 241 sea pens characterised by a global good health, with neither necrosis nor mucus production and with their polyps mostly extended. Cluster 2 was the least represented (32 sea pens) and is described by colonies presenting visual signs of bad health, with the proportion of the colony affected by necrosis always higher or equal to 50%. Finally, cluster 3 was constituted of 112 sea pens characterized by an elevated mucus production, the absence of associated fauna, contraction of polyps and lower levels of necrosis (*i.e.* up to 25% of the colony). Cluster 1 was separated from cluster 2 and 3 the first axis of the MCA while cluster 2 appeared to be separated from the two others within second axis (Fig. 3). While depth did not significantly explain this clustering (Student’s t-test, eta2 = 0.01; p-value = 0.15), distance from the farm was a significant factor (Student’s t-test, eta2 = 0.17; p-value < 0.001). Indeed, the average distance of sea pens from the farm of cluster 1 and 2 was significantly different from the average distance from the farm for the entire sea pen population, all clusters combined (Table 3). Particularly, cluster 1 was associated with higher distance from farm (mean distance from farm of 1262 m) while cluster 2 was associated with lower distance from farm (mean distance from farm of 721 m; Table 3).

**Table 3:** Mean distance from farm ± standard error of the *Virgularia mirabilis* colonies described for each cluster (see Figs. 3 and 4 for clusters definition).

Cluster	Mean distance from farm (m)	V test correlation	p-value
1	1262 ± 30	7.9	**2.5 10**^**–15**^
2	906 ± 89	− 1.7	0.09
3	721 ± 70	− 7.4	**1.2 10**^**–13**^

V-test correlation value can be seen as a “normalized” difference between the mean distance from farm for a clusters and the overall mean all clusters combined. Positive value signifies that mean distance from farm of colonies from this cluster is greater than the distance from farm of all colonies all clusters combined. p-value represent significatively of the Student’s t-test evaluating the correlation between the clusters and the distance from farm variable (see Husson et al.^40^).

Significant values are in bold.

**Figure 5:**
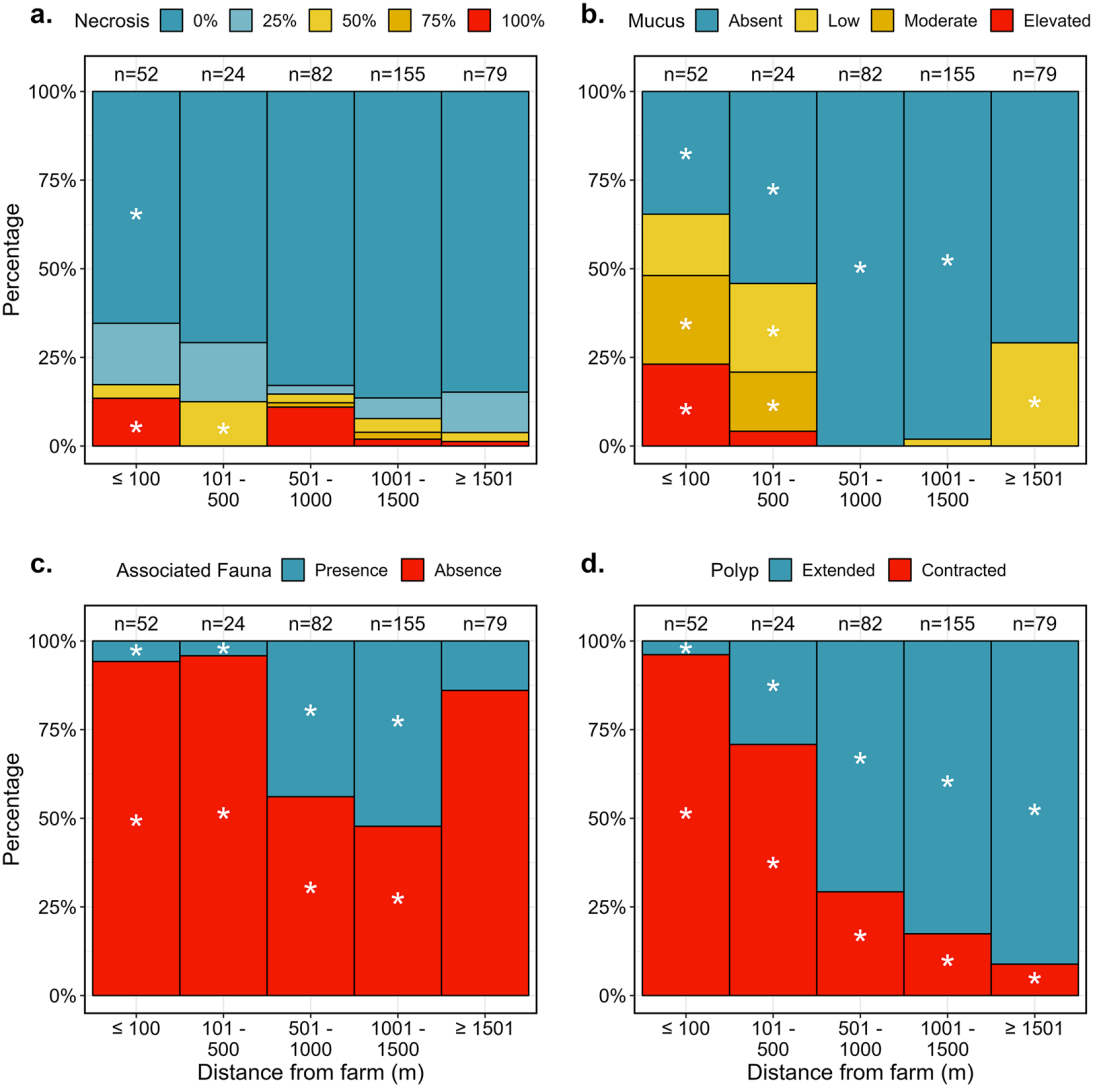
Proportion of *Virgularia mirabilis* exhibiting different levels of necrosis (**a**), mucus production (**b**), presence of associated fauna (**c**) and polyps’ positions (**d**) according to the distance from farm. White star represents significant differences between the proportion of a modality at a given distance and the mean proportion of this modality all distance combined.

The distance from the farm was a significant explanatory factor of the level of necrosis in *V. mirabilis* colonies (Chi-square test, χ^2^ = 67.79, p-value < 0.001; Fig. 5a). Indeed, there was a significantly higher proportion of colonies exhibiting 100% necrosis closer than 100 m from the farm, as well as a lower proportion of colonies exhibiting 0% necrosis (Fig. 5a). Sea pens exhibiting necrosis of 50% of their tissue were also significantly higher for colonies located between 101 and 500 m from the farm (Fig. 5a). Mucus production decreased significantly with the distance from the farm (Chi-square test, χ^2^ = 232.47, p-value < 0.001; Fig. 5b). While the proportion of sea pens that did not produce mucus was significantly lower within 500 m from the farm, the proportion producing a moderate amount of mucus was significantly higher within 500 m and the proportion of those producing an elevated amount of mucus was significantly higher within 100 m from the farm (Fig. 5b). Interestingly, we also observed a proportion (approximately 25%) of sea pens with low production of mucus above 1500 m from the farm. The presence of associated fauna also significantly changed with the distance from the farm (Chi-square test, χ^2^ = 110.84, p-value < 0.001; Fig. 5c). Within 500 m from the farm, associated fauna was absent in most of the sea pens (approximately 95% of the sea pens did not exhibit any associated fauna) while between 501 and 1500 m from the farm, half of the sea pens had associated fauna (Fig. 5c). Additionally, the position of polyps clearly shifted from contracted to extended when moving away from the farm (Chi-square test, χ^2^ = 226.62, p-value < 0.001; Fig. 5d). 96% of the sea pens within 100 m from the farm had contracted polyps while 91% of the sea pens had extended polyps at distance greater than 1501 m from the farm (Fig. 5d).

## Discussion

Here we showed that the presence of the fish farm impacted the health state of *V. mirabilis* colonies in many different ways including: (1) a mucus overproduction, (2) tissue necrosis, (3) contraction of polyps’ tentacles and (4) the disappearance of associated fauna.

Deleterious effects of particle sedimentation have been recorded for both tropical^41,42^ and cold-water corals^18,43–45^. When exposed to enhanced particle sedimentation, many coral species use mucus secretion to clean their surface tissue from particles^42–44,46^. This reduces the risk of anoxic conditions development causing tissue damage and loss^46^. However, an overproduction of mucus may result in energy loss for the corals^46,47^ and may increase their susceptibility to viral infections^48,49^. Also, coral overburdening by POM, or by their own overproduction of mucus, may influence polyp’s activity, including their contraction, reducing feeding activity^19,43^, preventing the compensation of energy loss induced by mucus secretion. In the most severe case, high sedimentation rates may result in tissue necrosis, and complete colony mortality^19,41^.

**Figure 6:**
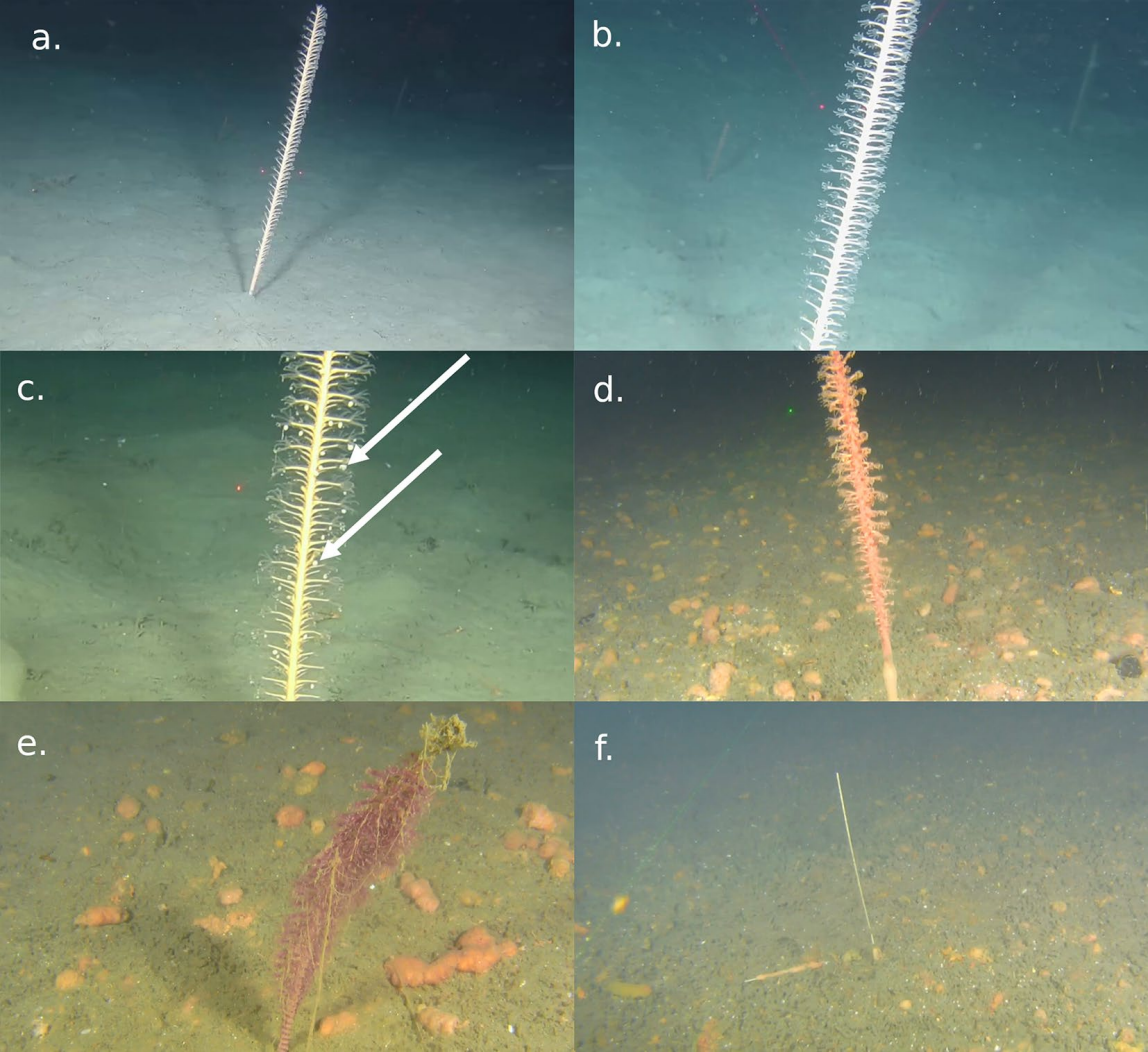
Selection of snapshots from the video transects (distance between two laser points = 10 cm). (**a**) A *Virgularia mirabilis* colony with a global good health. Colony located in transect A at 1370 m from farm. (**b**) Close up view of the colony of picture A with a focus on the polyps that are extended. (**c**) Close up view of a *V. mirabilis* colony with a global good health and harbouring several amphipods (examples showed by white arrows). Colony located in transect A at 900 m from the farm. (**d**) *V. mirabilis* colony producing a low amount of mucus. Fish faeces are visible on the seafloor surrounding the colony. Colony located in transect C at 17 m from the farm. (**e**) *V. mirabilis* colony producing an elevated amount of mucus and with its polyps contracted. Fish faeces are here also visible on the seafloor surrounding the colony. Colony located in transect C at 20 m from the farm. (**f**) Dead *V. mirabilis* colony with only the calcareous skeleton remaining (center) and another colony in poor condition, laying on the sediment, to the left. Fish faeces are here also visible on the seafloor surrounding the colony. Colony located in transect C at 60 m from the farm.

It is therefore likely that the poor health of sea pens observed in the vicinity of the studied farm is related to POM sedimentation coming from the fish farm. Indeed, salmon fish farms release considerable amounts of organic wastes and nutrients in the environment, generated from remains of uneaten food, fish feces and excretion^3,6,8,50^. Thus, close to aquaculture localities, POM sedimentation can reach eight to 20 times the natural sedimentation rate^11,13^. The environmental footprint of these effluents depends on several factors, *e.g*. hydrodynamic properties, depth, and size of the farm^12^. Typically, the strongest environmental footprint caused by a fish farm is estimated to extend 40–150 m away from the farm^11^. But in more dispersive area, this footprint can be greater, reaching 550 m^13^ to 600 m^11^. Our present results suggest that sites located further than 680 m away from the farm are not exposed to significant amounts of effluents generated by the farm, at this stage of the production cycle. Although this study did not allow a characterization of the sedimentation directly underneath the farm, the dispersion model (Fig. 2) and the underwater videos (Fig. 6) confirmed the presence of fish faeces on the seafloor and suggested that the environmental footprint is the strongest to a distance of 200 m from the farm. The deposition of fish faeces probably initiated an active cleaning response (*i.e.* enhanced mucus production) in the sea pens located within the footprint of the farm. The decrease in polyp activity, observed in the same area, could be caused by mucus overproduction, covering the sea pens, and preventing their normal functioning. It also appears that when sedimentation thresholds are overpassed, permanent damage can result. Indeed, the most affected sea pens showed signs of tissue necrosis and in some cases complete colony mortality. The present results do not provide an explanation regarding the shift from altered behavioural response (i.e. mucus overproduction and decrease of polyp activity) to permanent tissue damages. This may be explained by exceeded thresholds in terms of (1) duration of exposure to effluents from the farm, (2) amount of POM present in the environment or (3) a combination of both.

The community of mobile taxa associated with sea pen fields is normally rich^35,37^. Here, sea pens affected by the fish farm seem to present less associated fauna than sea pens further away from the farm. These confirm observations that the mobile fauna associated with corals (mainly crustaceans) can be negatively affected by deteriorating health of their host^51–54^. Interestingly, poor health condition of sea pen close to the farm didn’t trigger colonisation of the sea pen skeleton by epibionts. This contrasts with the results of previous studies, showing that the presence of sessile associated taxa on corals can be an efficient proxy of poor health condition, as these taxa can colonise damaged tissue and bare skeleton^55–57^. However, the majority of the sessile taxa considered in the previously cited studies are algae and the results are therefore not applicable to our study site that is fully in aphotic conditions. Further work is needed for a better understanding of processes involving sea pens health and their associated community, using more holistic approaches including both qualitative (*i.e.* identification of the taxa) and quantitative (*i.e.* count of individuals and/or estimation of the coverage) investigations of the associated communities. In addition, it would be important to assess the health state of this associated community, that may be impacted by the enhanced organic loading in the same way as the sea pens.

Finally, our results showed that *V. mirabilis* in close proximity to fish farm incorporate into their tissue emamectin B1a (*i.e.* marker residue of EMB) released into the envrionment during and following sealice treament at the farm. Also, depuration seems to be slow as emamectin B1a was still present in sea pens tissues 56 days after the treatment. It is noteworthy that while EMB residue was present in sea pens tissue, it is highly unlikely that this chemotherapeutant plays a significant role in the poor health state observed compared to POM effluents. Indeed, a laboratory experiment on the impact of EMB on another Pennatuloidea species (*Pennatula phosphorea*) didn’t show any signs of toxicity which suggests that specific binding sites targeted by EMB may not be present in Pennatuloidea^33^. Persistence of EMB in fish tissues is an important property for its use as a veterinary medicine as it allows long-term protection against sea lice^58^. Indeed, EMB has been showed to persist in liver and kidneys of Atlantic salmon for 90 days after administration^24,59^. Thus, excretion and defecation of Atlantic salmons will contain EMB throughout this period, involving an EMB exposure of the surrounding area longer than the treatment of the target species itself. The presence of EMB residue in the tissue of *V. mirabilis* is consistent with the results of a laboratory experiment that also measured levels of emamectin B1a in tissue of the sea pen *Pennatula phosphorea* after an exposure^33^. Emamectin B1a concentrations up to 0.091 ± 0.012 µg mgDW^−1^ were measured in *P. phosphorea* tissues. This concentration is approximately 100 times higher than the concentration we measured in the present study, however, the EMB concentration in seawater used for this exposure experiment (0.8 mg L^−1^) was much higher than concentrations that could exist in situ (*e.g.* 4.16 × 10^–6^ μg L^−1^ according to models Willis and Ling^28^). Moreover, measurements of EMB residue in the tissue were done about 6 days after the exposure while in the present study the sea pens were sampled 56 days after the EMB treatment. Telfer et al.^60^ detected concentrations up to 5 µg kg^−1^ wet weight in various benthic species (*i.e. Pagurus* spp., *Carcinus maenas*, *Munida rugose*, *Asterias rubens*, *Mytilus edulis* and *Buccinum undatum*), sampled at approximately 100 m from a salmon farm treated with EMB in Scotland. These results are of the same order of magnitude as the concentrations measured in *V. mirabilis* in the present study. Furthermore, Telfer et al.^60^ detected quantifiable levels of EMB up to 1-month post-treatment (in *C. maenas* and *Pagurus* spp.), and no EMB could be detected in tissues 4-month post-treatment. It suggests that depuration of EMB residue to levels below the limit of quantification for these species occurred at a time between 1 and 4 months, which may be consistent with the results of our study. In further studies it will be important to assess the persistence of EMB residue in the tissue of *V. mirabilis* and estimate more accurately the time needed for complete depuration. The persistence of EMB residue in *V. mirabilis* tissues may have major implications for the species in case of multiple sea lice treatments, potentially leading to enhanced bioaccumulation of this compound. Furthermore, this persistence of EMB in *V. mirabilis* can potentially lead to scaling effects within the food web driven by processes such as bioaccumulation of biomagnification, as sea pens are preyed by a variety of species^61^ such as asteroids and nudibranchs (see Supplementary Fig. S2).

## Conclusion

Here we showed (1) the presence and persistence of emamectin B1a in *V. mirabilis* tissues 56 days after a sea-lice treatment, as well as (2) lethal and sublethal effects caused by effluents released from an Atlantic salmon farm on the sea pen *V. mirabilis*. The persistence of EMB in tissues indicates a potential for bioaccumulation and scaling effects in the food web that should be carefully examined in further studies. Concerning impacts of organic effluents, sea pens exhibited clear signs of mucus overproduction visible up to 500 m from the fish farm while sea pens very close and directly beneath the farm exhibited a high degree of tissue necrosis and in the most severe case complete tissue loss and mortality. As a precautionary measure to mitigate these impacts, we strongly recommend implementing rigorous site selection criteria to avoid significant sea pen fields when establishing new fish farming facilities.

Nevertheless, it is noteworthy that *V. mirabilis* colonies, despite their poor health state, were still present underneath the farm although this location has been used for farming for over twenty years. This suggests that *V. mirabilis* population possesses the capacity to recover from the impacts from the farm, thereby preventing complete disappearance from the area. In this context, it would be particularly important to run a temporal survey following the health state of the sea pen population during an entire production cycle including during and after the post-production fallowing period.

## Methods

### Study site

The study site is an Atlantic salmon fish farm located in Boknafjord in Rogaland County, southern Norway. The farm has been in activity since August 2000, currently constitutes of 13 open-sea cages rings (50 m in diameter) and with a maximum allowable biomass of 7020 tonnes. After being fallowed for five months, the farm started a new production cycle in July 2022. During our survey in February and March 2023 the biomass of fish was approximately half of the maximum biomass capacity of the farm. In order to face sea-lice infestations, the farm used an emamectin benzoate infeed treatment between 28/12/2022 and 06/01/2023. Three different study transects (A, B and C) were defined based on pre-existing knowledge from bathymetric data and habitat predictions^62^ (Fig. 1). Transect A is approximately 1400 m long with a north-west south-east orientation, transect B is approximately 270 m long with a south-west north-east orientation. The two transects converge at their eastern extremity, which is 680 m away from the farm. Transect C is approximately 400 m long with a north south orientation. About 180 m of its southern extremity is located directly below the fish farm. The depth remained quite stable along the different transects up to 1400 m away from the farm (between 170 and 190 m deep) before dropping to 240 m at 1740 m from the farm. The distance from the farm has been calculated from the center of the cage the closest to the transects.

### Video survey and annotation

The three transects were surveyed with an Argus mini ROV (Remotely Operated Vehicle), equipped with two powerful LED lights and HD camera, from research vessel *K. Bonnevie*. For each transect, the ROV was kept as close to the sea bottom as possible. When a *V. mirabilis* colony was spotted, the ROV was stopped shortly to obtain a close-up view. The position of the ROV was continuously recorded during each transect using a cNODE transponder and HiPAP system (Kongsberg). Transect A was surveyed on 03/02/2023, transect B on 07/02/2023 and transect C on 03/03/2023.

Video were annotated a posteriori using VideoNavigator 2.1.33.00, a software for annotation of benthic videos developed by the Institute of Marine Research, Norway. All video transects has been annotated by a single observer. When a sea pen was observed on the video, its presence was recorded, along with different information on its health state and morphology (*i.e*. presence of necrosis, mucus production, presence of associated fauna and polyps’ position; Fig. 6). The criteria considered for each sea pen are fully described in Table 4. Note that in the present study, associated fauna defines all animals that found living space, food and/or shelter on sea pens. Thus, both the attached (most of the time referred as “epibionts”, *e.g.* hydroids, bryozoans) and free living (*e.g*. shrimps, amphipods) taxa were pooled. Each described sea pen was geographically localised along the transects using the underwater positioning tracking.

**Table 4:** List of the health and morphology categories with associated modalities and abbreviations that were assigned to *Virgularia mirabilis* colonies during video transects analysis.

Category	Modalities	Abbreviation
Polyps	Extended	Pol_Ext
	Contracted	Pol_Cont
Associated fauna	Absence	AF_Abs
	Presence	AF_Pres
Mucus production	Absence	Muc_Abs
	Low production	Muc_Low
	Moderate production	Muc_Mod
	Elevated production	Muc_Elev
Necrosis	0% of the colony	Necr_0%
	25% of the colony	Necr_25%
	50% of the colony	Necr_50%
	75% of the colony	Necr_75%
	100% of the colony	Necr_100%

### Site characterisation

Sediment and seawater were sampled at five different locations along the transects A (locations A1, A2, A3, and A4, respectively at 2110 m, 1200 m, 940 m, and 680 m from the farm; Fig. 1) and B (location B1, 790 m away from the farm; Fig. 1). No sample was taken close to transect C due to the presence of anchoring lines in close proximity.

Sediment was sampled to measure (1) organic content and (2) EMB concentration. To do so, surface sediment was sampled using a Van Veen grab (0.1 m^2^) at each of the five locations (n = 3 for each location). Sediment was directly transferred into two different 50 mL Falcon tubes and stored in − 20 °C freezer pending analyses. The first tube was used to measure the proportion of organic content in sediment as follows: sediment was dried to a constant weight (60 °C, 5 days), weighed (giving dry weight), and burned in a muffle furnace (450 °C, 6 h) and reweighed (giving ash free dry weight). Proportion of organic content in the sediment is then given in percentage as the ratio between the loss of ignition (dry weight minus ash free dry weight) and initial dry weight. The second tube was used to measure emamectin B1a concentration in sediment according to the method described in part 2.6.

Seawater was sampled to measure concentration of (1) nutrients, (2) ammonia and (3) Particulate Organic Matter (POM). At the same five locations, bottom seawater was sample (1 m above the seafloor) using Niskin bottles (n = 3 for each location). For nutrient measurements, 20 mL of seawater was immediately transferred to scintillation vials and preserved with two drops of chloroform before being stored in a fridge pending analyses. Using a Skalar autoanalyzer, nitrite (NO_2_^−^, µmol L^−1^), nitrate (NO_3_^−^, µmol L^−1^), phosphate (PO_4_^3−^, µmol L^−1^) and silicate (SiO_4_^4−^, µmol L^−1^) concentrations were measured. Ammonia samples have been stored frozen until fluorometric determination of ammonium by direct segmented flow analysis (*Alpkem Flow Solution IV* autoanalyzer: Kérouel and Aminot^63^; Holmes et al.^64^). Regarding POM concentration, 2 L of seawater was filtered onboard immediately after sampling under gentle vacuum onto burned and pre-weighed Whatman GF/F filters (25 mm diameter). Each filter was then dried to a constant weight (45 °C, 1 day), weighed, and burned in a muffle furnace (450 °C, 5 h) and reweighed in order to calculate ash free dry weight and POM concentration (in g L^−1^).

### Dispersion model

The model setup is similar to the one used by Carvajalino-Fernández et al.^65^. The Regional Ocean Modelling System (ROMS^66^) was used to model ocean currents in the vicinity of the farm. A refined version of the model framework NorKyst800^67^ was used in a region (130 km × 180 km) around the farm, with a horizontal resolution of 80 m. The original NorKyst800 model was used on the boundary. NorKyst800 has been validated by a wide range of hydrodynamic measurements and has been shown to simulate realistic currents within Norwegian fjords^68,69^. Dispersion of organic matter from the farm was modelled using the particle transport model LADiM (https://github.com/pnsaevik/ladim). Actual feed data from the fish farm was used to initialize the particle field, with a feed-to-POM conversion factor of 24%^70^. Sinking velocities were taken from Bannister et al.^71^. Fish farm cage locations were extracted from aerial photos. The particles were emitted at a depth of 20 m, in the period between 01/08/2022 and 07/01/2023.

### Sampling of *V. mirabilis* colonies

Sea pens were sampled at different distance from the farm to measure the concentration of EMB in the tissue. Three colonies were sampled at sites A1, A2, A3 and A4 (respectively 2110 m, 1200 m, 940 m, and 680 m away from the farm) on 03/02/2023 and five colonies were sampled at site C1 (directly below the farm) on 03/03/2023, using the manipulator arm of the ROV. Exact coordinates of each seas pen are available in Supplementary Fig. S3. Calcium carbonate skeleton the sea pens was carefully removed, using disposable gloves, and carefully cleaned dissecting equipment, remaining tissue has been transferred into 50 mL Falcon tube and stored in − 20 °C freezer pending emamectin analyses described in part 5.6.

### Emamectin analysis

Sea pens tissues and sediment samples were homogenized and weighed before they were freeze-dried for 72 h, the first 24 h at -50 °C followed by 48 h at 25 °C, at 0.2–0.01 mbar (Labconco Freezone). Thereafter the samples were once again weighed and homogenised. Dry weight (DW) was calculated based on the difference in weight of the samples before and after freeze-drying. Residues of EMB was measured as emamectin B1a, which is the main component of emamectin^72^ (≥ 90%) and the marker residue for EMB after use at fish farms^39^. For analyses of sediment samples, 1.00 ± 0.05 g DW were weighed into plastic tubes. Concerning *V. mirabilis,* the tissue available was limited, therefore, the whole sea pen was weighed into plastic tubes, the weight varied from 0.0179 to 1.0287 g DW. A six-point calibration curve was prepared by adding increasing amount of EMB (Sigma Aldrich, Germany) to vials without matrix. The amount EMB added corresponds to concentrations ranging from 2.0 to 100 µg emamectin B1a kg^−1^. For quality control a blank sample of salmon (salmon not treated with EMB), a procedural blank and two quality control samples (salmon with known concentrations; 2.0 and 85 µg emamectin B1a kg^−1^), were included. Stable isotope labelled internal standard, emamectin-d3 (Toronto Research Chemicals, Canada) was added to all samples. Acetonitrile was added for extraction, thereafter the samples were shaken for 10 min, followed by 10 min sonication. The extracts were transferred to new vials and concentrated at 50 °C under nitrogen flow. The residues were dissolved in methanol/water (80/20) and filtered through a 0.45-μm filter. Analysis was performed by an Agilent 1260 LC-system (Agilent Technologies, Waldbronn, Germany) coupled to an Agilent 6460 triple quadrupole mass spectrometer (Agilent Technologies, Waldbronn, Germany). The instrument was equipped with an ESI jet stream source operated in positive mode. The samples were injected to a reverse-phase Zorbax C18 RRHD analytical column 2.1 mm × 50 mm, 1.8 μm (Agilent Technologies, Waldbronn, Germany). The mobile phase consisted of methanol and 0.1% formic acid in water, with a flow rate of 0.5 mL min^−1^. Chromatography was performed according to a stepwise gradient: 0–0.2 min, 10% methanol; 3.3–3.5 min, 90% methanol; 3.6–5 min, 10% methanol. All gradient steps were linear. The ESI source was operated in positive mode with drying gas temperature 200 °C; gas flow 6 L min^−1^; sheath gas heather 400 °C; sheath gas flow 12 L min^−1^; nebuliser pressure 35 psi. Detection and quantification were conducted using multiple reaction monitoring (MRM) of the following transitions: emamectin B1a, 886.3 m/z → 158.1 m/z (quantifier) and 886.3 m/z → 82.2 m/z (qualifier); emamectin-d3, 889.3 m/z → 161.1 The limit of quantification (LOQ) was 1.0 µg kg^−1^ dry weight (DW) for the sediment samples. For the sea pens, due to the variation in sample weight, the LOQ varied from 1.0 to 20 µg kg^−1^DW. The method was linear in the range analysed (up to 100 µg kg^−1^), and inter-run precision was < 20%.

### Statistical analyses

Multiple correspondence analysis (MCA^73^) was used to visualise the distribution of sea pens within the different health and morphology categories (*i.e.* presence of necrosis, mucus production, presence of associated fauna and polyps’ position). MCA is an extension of the correspondence analysis that allows coordination among several categorical variables^74^. Depth and distance from farm of each sea pen has been added to the MCA as supplementary quantitative variable. To define clusters of sea pens that shared similar patterns of health and morphological traits, a hierarchical cluster analysis using Ward linkage^75^ was performed based on the Euclidian distance of the sea pen coordinates along the first two MCA axes. In order to investigate if clusters are significantly correlated with the quantitative variable “depth” and “distance from farm” Student's t-test was used^40^. Pearson Chi-square (χ^2^) homogeneity tests were used to investigate significant differences of mucus production and necrosis according to the distance from the farm. Based on residuals of this test, post-hoc analysis using Bonferroni correction has been applied. The statistical analyses were performed using RStudio (V2023.03.0^76^) with the packages *FactoMineR*^77^ and *ggplot2*^78^.

### Ethic statement

The research was conducted according to relevant guidelines and regulations.

### Informed consent

The authors would like to confirm that the company operating the Atlantic salmon farm studied here has given its consent for this experiment to be carried out, on condition that the name of the farm and the company, as well as the exact location of the farm, are not made public.

### Supplementary Information

Supplementary Figures.

## Data Availability

The datasets are available from the corresponding author upon request.
