# Supplementary material for: Effects of aquaculture effluents on the slender sea pen Virgularia mirabilis
Source: Sci Rep. 2024 Apr 24;14:9385. doi: 10.1038/s41598-024-59613-3 (PMC11039756; doi:10.1038/s41598-024-59613-3)

Effects of aquaculture effluents on the slender sea pen *Virgularia mirabilis* - Bastien Taormina *et al.* 2024

Supplementary Figure S1: Location of the 385 *Virgularia mirabilis* colonies described in this study. Each point represents a colony (Map created by author using QGIS 3.22.7 software: https://qgis.org/).

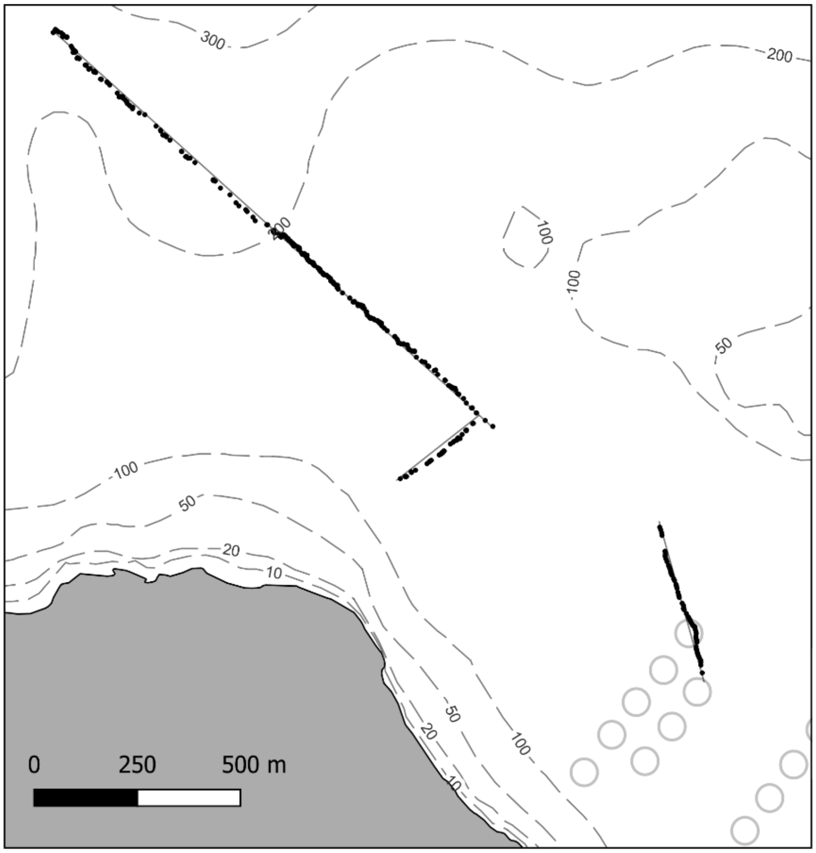

Supplementary Figure S2: Snapshot of video transect A showing a *Virgularia mirabilis* colony predated by a sea star (*Hippasteria phrygiana*). Observed 1,350 m away from the farm.

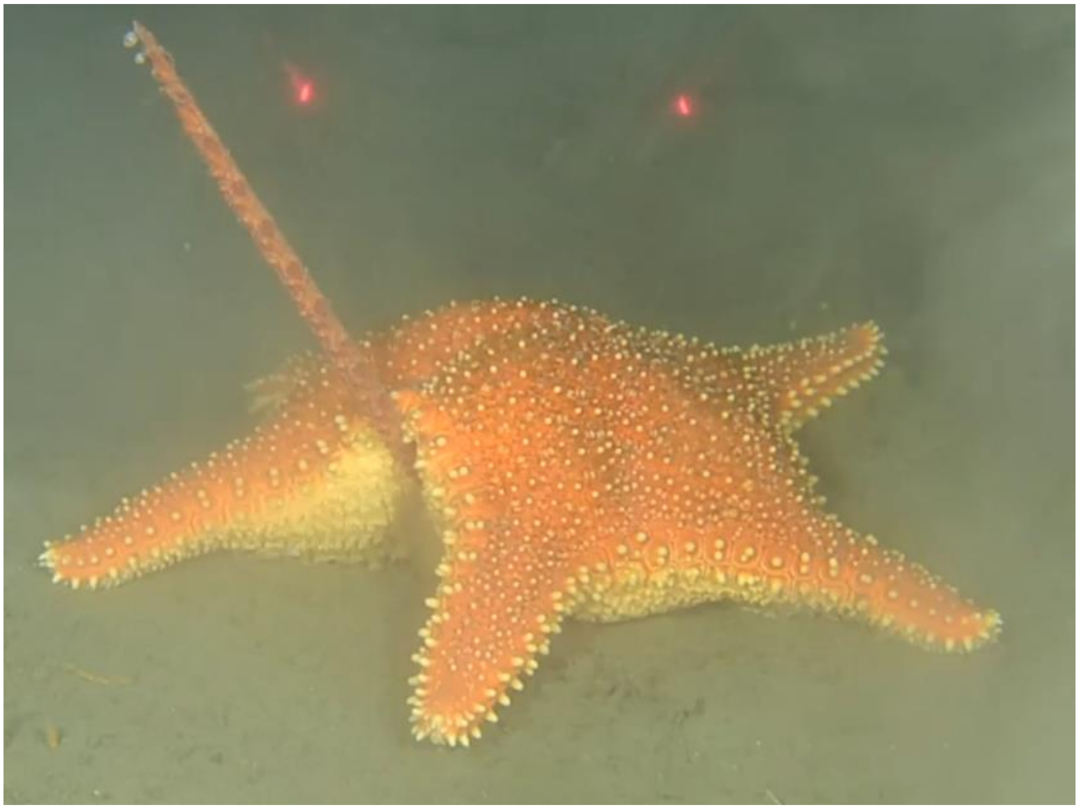

Supplementary Figure S3: Locations of the *Virgularia mirabilis* colonies sampled for emamectin benzoate measurement in tissue (Map created by author using QGIS 3.22.7 software: https://qgis.org/).

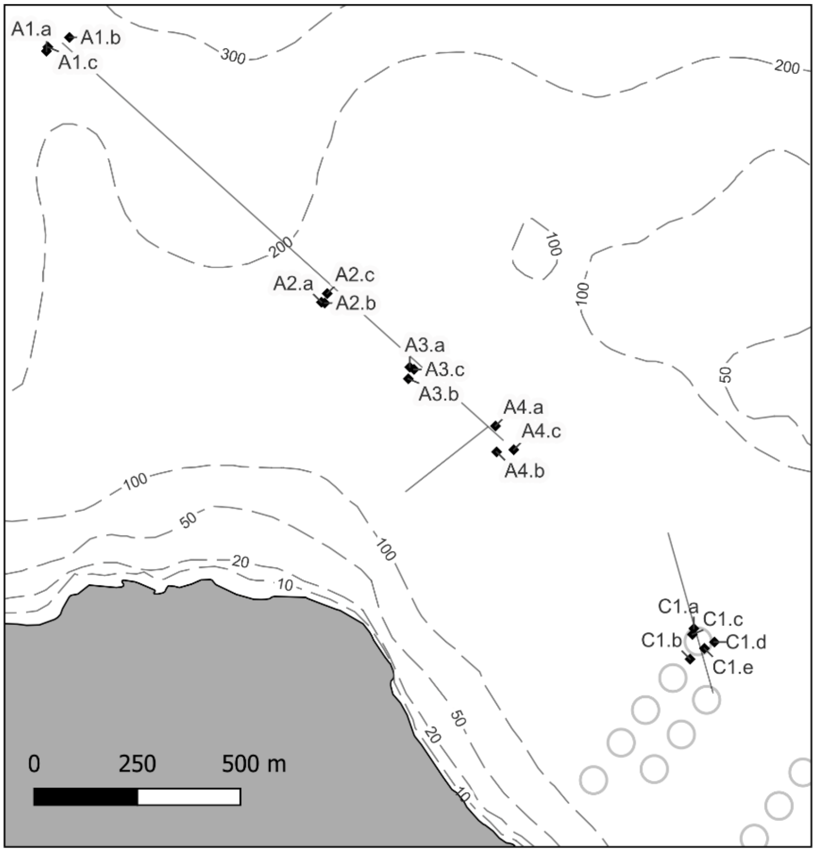

Supplement: Supplementary file 1 — Supplementary Figures. [file 41598_2024_59613_MOESM1_ESM.pdf]
